# Variability in infection dynamics emerges from the interplay between unique host and pathogen characteristics

**DOI:** 10.1038/s41598-025-01351-1

**Published:** 2025-06-02

**Authors:** Ruth Rodríguez-Pastor, Mario Garrido, Nadav Knossow, Naama Shahar, Ron Flatau, Hadas Hawlena

**Affiliations:** 1 Mitrani Department of Desert Ecology, Swiss Institute for Dryland Environmental and Energy Research, The Jacob Blaustein Institutes for Desert Research, Ben-Gurion University of the Negev, Midreshet Ben-Gurion, Israel; 2 Present Address: Agri-Food Institute of Aragon (IA2)-University of Zaragoza-CITA, Zaragoza, Spain; 3 Present Address: Department of Biology and Geology, Physics and Inorganic Chemistry, Biodiversity and Conservation Area, Rey Juan Carlos University, Móstoles, Madrid, Spain; 4 Present Address: Ocean Genome Legacy Center, Northeastern University, Massachusetts, USA

**Keywords:** Host heterogeneity, Inoculations, Infection dynamics, Multiple hosts, Multiple parasites, Within-host dynamics, Community ecology, Ecological epidemiology, Microbial ecology

## Abstract

Variation in infection dynamics across host species can profoundly influence parasite epidemiology, ecology, and evolution. However, because experiments involving multiple hosts and multiple parasite species that capture the full infection dynamics within hosts are rare, the underlying mechanisms of host heterogeneity effects across different parasite species remain largely unknown. We dissected the specific roles of host and pathogen effects in shaping within-host infection dynamics using a model system comprising three coexisting rodent species from Israel’s northwestern Negev Desert and their predominant bacterial pathogens, *Bartonella krasnovii* A2 and *Mycoplasma haemomuris*-like bacterium. To test whether the effects of host species heterogeneity on parasite dynamics mainly derive from host trait variation (“host trait variation hypothesis”) or rather reflect a unique host-parasite interaction (“specific host-parasite interaction” hypotheses), we inoculated rodents of the three species (*Gerbillus andersoni*, *G. pyramidum*, and *G. gerbillus*) with either *Bartonella* or *Mycoplasma* and compared their infection dynamics during primary infection and upon reinfection. According to the “host trait variation” hypothesis, host heterogeneity effects would be consistent across parasite species. Supporting this hypothesis, both bacterial pathogens exhibited reduced performance in *G. gerbillus* compared to the other rodent species. However, consistent with the alternative “specific host-parasite interaction” hypothesis, all other aspects of the infection dynamics of these two pathogens exhibited varying trends across the three hosts, indicating the uniqueness of each pathogen-host interaction. Our findings support the notion that the variability in infection dynamics is not solely attributable to host heterogeneity, but rather emerges from the interplay between specific host characteristics and parasite traits. We discuss potential directions for future research to deepen our understanding of within-host dynamics in multispecies communities. We stress the necessity of analyzing each distinct host-parasite interaction independently when making inferences about community patterns based on infection dynamics within hosts until more experimental data on natural systems are accumulated.

**Supplementary Information:**

The online version contains supplementary material available at 10.1038/s41598-025-01351-1.

## Introduction

In nature, parasites—organisms that live in or on a host and extract resources at the host’s expense—show considerable variation in their infection dynamics across different host species. For example, growth rates, infection duration, infection loads, and even repeated infection patterns of the same parasite species can vary across different hosts^1–3^. Because this host heterogeneity can profoundly influence parasite epidemiology, ecology, and evolution^4,5^, it is essential to understand the factors contributing to these variations in infection dynamics.

An increasing number of field surveys and host-parasite incompatibility experiments provide evidence of the interaction between host characteristics and parasite traits in shaping host-parasite dynamics ^e.g.,6–10^. To better understand the mechanisms behind the variability in within-host infection dynamics, high-resolution infection experiments have been conducted. One line of experiments focuses on the dynamics of a single parasite across different host types (e.g., species, strains, age groups). These experiments show that variation in host traits, such as age, sex, body mass, and immune response, can explain differences in infection dynamics between hosts^11–14^. Another line of experiments examines how different parasites infect a single host type. These studies suggest that different parasite species or strains may provoke distinct host responses^15,16^. However, due to the logistical challenges of fully characterizing infection dynamics across multiple parasites and host types that mirror natural communities, such studies are rare and mostly limited to invertebrate hosts, which lack the immune complexity of vertebrate hosts^17^.

The rodent-bacteria communities in the dunes of Israel’s northwestern Negev Desert offer a promising model system to complement these studies and dissect the specific roles of host and parasite effects in shaping within-host infection dynamics. This region features distinct areas hosting rodent communities with varying species compositions in proximity to each other, with the most common species being *Gerbillus andersoni*, *Gerbillus gerbillus*, and *Gerbillus pyramidum*. The prevalent bacterial pathogens found in these rodents’ blood are *Mycoplasma haemomuris*-like bacterium and species of *Bartonella*^18,19^ (for species-specific prevalences, see Supplementary Fig. S1). Although the three closely related rodent species share traits, such as being nocturnal, psammophilic (adapted to sandy environments), and granivores, living in burrows, and facing similar pressures from ectoparasites and predators^20–22^, there is a notable difference between them in terms of their interaction with *Mycoplasma*. Specifically, *G. andersoni* acts as a *Mycoplasma* amplifier, while the other two act as *Mycoplasma* diluters^2^. Thus, if the impact of host heterogeneity on parasite dynamics primarily stems from variations in host traits (“host trait variation” hypothesis), we expect, for the *Bartonella* pathogens, a pattern similar to *Mycoplasma* infections. Specifically, we expect that *Bartonella* infection will persist longest in *G. andersoni* hosts, while the infection duration will be shorter and similar in the other two rodent species^2^.

On the contrary, although both *Mycoplasma* and *Bartonella* are bacterial pathogens targeting host red blood cells (RBCs) and multiply within the vascular system, they belong to phylogenetically distant genera and exhibit differences in their life history strategies (detailed in the “*Study organisms*” section below). Therefore, if infection dynamics reflect specific host-parasite interactions (“specific host-parasite interaction” hypothesis), we anticipate observing distinct patterns of host specialization between the two pathogens. For instance, the natural distribution of *Bartonella* among the three rodent species suggests that this bacterium perceives the rodents to be similar^19^. Based on this observation, we expect to observe similar *Bartonella* dynamics in all three rodent species.

To distinguish between the two hypotheses, we inoculated males from each of the three rodent species with either *Bartonella krasnovii* A2 or *Mycoplasma haemomuris*-like bacterium. We then quantified the infection dynamics during primary infection and after reinfection. As predicted by the “host trait variation” hypothesis, both *Bartonella* and *Mycoplasma* pathogens showed reduced performance in *G. gerbillus* compared to the other rodent species. However, in line with the “specific host-parasite interaction” hypothesis, all other aspects of the infection dynamics of these two blood-related pathogens exhibited varying trends across the three hosts. These findings support the notion that the variability in infection dynamics across hosts may be influenced by the specific interactions between host and parasite types. Thus, to gain a deeper understanding of epidemiological dynamics within multispecies communities in natural settings, it is crucial to examine the infection dynamics of each host-parasite interaction individually. This approach should complement our understanding of parasite-specific interactions with their hosts and coinfections.

## Methods

### Study organisms

*G. andersoni*, *G. gerbillus*, and *G. pyramidum* coexist within the sands of the northwestern Negev Desert in Israel^23,24^. The individual rodents utilized in our experiments originated from a laboratory colony maintained by Hawlena. This colony comprises descendants of wild rodents bred and raised in the laboratory environment for approximately eight years. These rodents have not been exposed to *Bartonella* or *Mycoplasma* species, nor have they undergone any form of drug treatment. The subjects selected for our study were non-reproductive adult males aged between 2 months and 3.5 years, with average body masses of 42.7 ± 1.10 g, 34.1 ± 0.807 g, and 65.2 ± 2.58 g for *G. andersoni*, *G. gerbillus*, and *G. pyramidum*, respectively. We chose to focus on non-reproductive adult males rather than sampling other intraspecies groups to minimize variability unrelated to the host and pathogen species. However, non-reproductive adult males may provide a reliable representation of the entire population of each species during the summer, when the dilution effect of *Mycoplasma* was observed. During this period, only non-reproductive adults are present, and both male and female hosts exhibit similar pathogen prevalences and intensities (H.H. et al., 2011, unpublished data). The animals were housed individually in plastic cages measuring 34 × 24 × 13 cm (total volume of 10,608 cm³) and containing a 1-cm layer of autoclaved sand. These cages were located within an animal facility maintained at an ambient temperature of 24.5 ± 1 °C and a photoperiod of 12 D: 12 L. The rodents were daily provided with millet seeds *ad libitum* as their primary food source and fresh alfalfa as their water source. During the experiment, only one *G. pyramidum* rodent was euthanized due to poor physical condition. The animal was placed in an induction chamber and exposed to a 20% CO_2_/O_2_ (v/v) mixture. Once the animal lost its righting reflex (indicating loss of consciousness), the CO_2_ concentration in the chamber was increased to 100% (v/v) and maintained until the animal ceased breathing.

*Bartonella* and *Mycoplasma* are the predominant bacterial pathogens within our study system^18,25^. *Bartonella* bacteria are mainly flea-borne, penetrating the RBCs and causing acute infections^26^. In the Negev region, they are clustered into four species and more than 30 strains^27,28^. The *Bartonella* strain utilized in our experiment was *B. krasnovii* A2, which was isolated from the blood of *G. andersoni* and belongs to the most prevalent lineage infecting rodents in this study system^27^. In contrast, *Mycoplasma* bacteria parasitize the RBC outer membrane (hemoplasmas^29^ and cause chronic infections^30)^. *Mycoplasma haemomuris*-like bacteria are uncultivable, and thus, hosts were inoculated with preserved blood from *Mycoplasma*-positive *G. andersoni*. In the Negev region, they are clustered into one strain, which is mostly transmitted through host-to-host contact^19,30^. This *Mycoplasma haemomuris*-like strain is hereafter designated as *Mycoplasma*. All the bacteria and rodents in this study were wild organisms, either preserved in the lab (bacteria) or bred and maintained in captivity (rodents). Neither of the bacterial species has undergone significant selection under lab conditions. The *Mycoplasma*-infected blood was collected from wild rodents in the field, preserved, and then inoculated into the rodents. Similarly, the *B. krasnovii* A2 was isolated from the blood of field-collected rodents, preserved, and subsequently inoculated into recipient rodents.

### Experimental design

At the beginning of each infection session (day 0), we inoculated five male specimens of each rodent species with either *Bartonella* or *Mycoplasma*. Notably, the *Mycoplasma* dynamics exhibited significant variability across individual hosts in terms of infection duration and likelihood of recurrence (Supplementary Fig. S2), unlike the relatively consistent behavior of *Bartonella* across individuals of the same host species (Supplementary Fig. S3). To further investigate this variability, we conducted a complementary infection session focusing exclusively on *Mycoplasma* by inoculating an additional six male hosts from each rodent species. Thus, over the course of the experiment, a total of 11 and five males of each species were inoculated with *Mycoplasma* and *Bartonella*, respectively (Supplementary Figs. S2−S3).

**Fig. 1:**
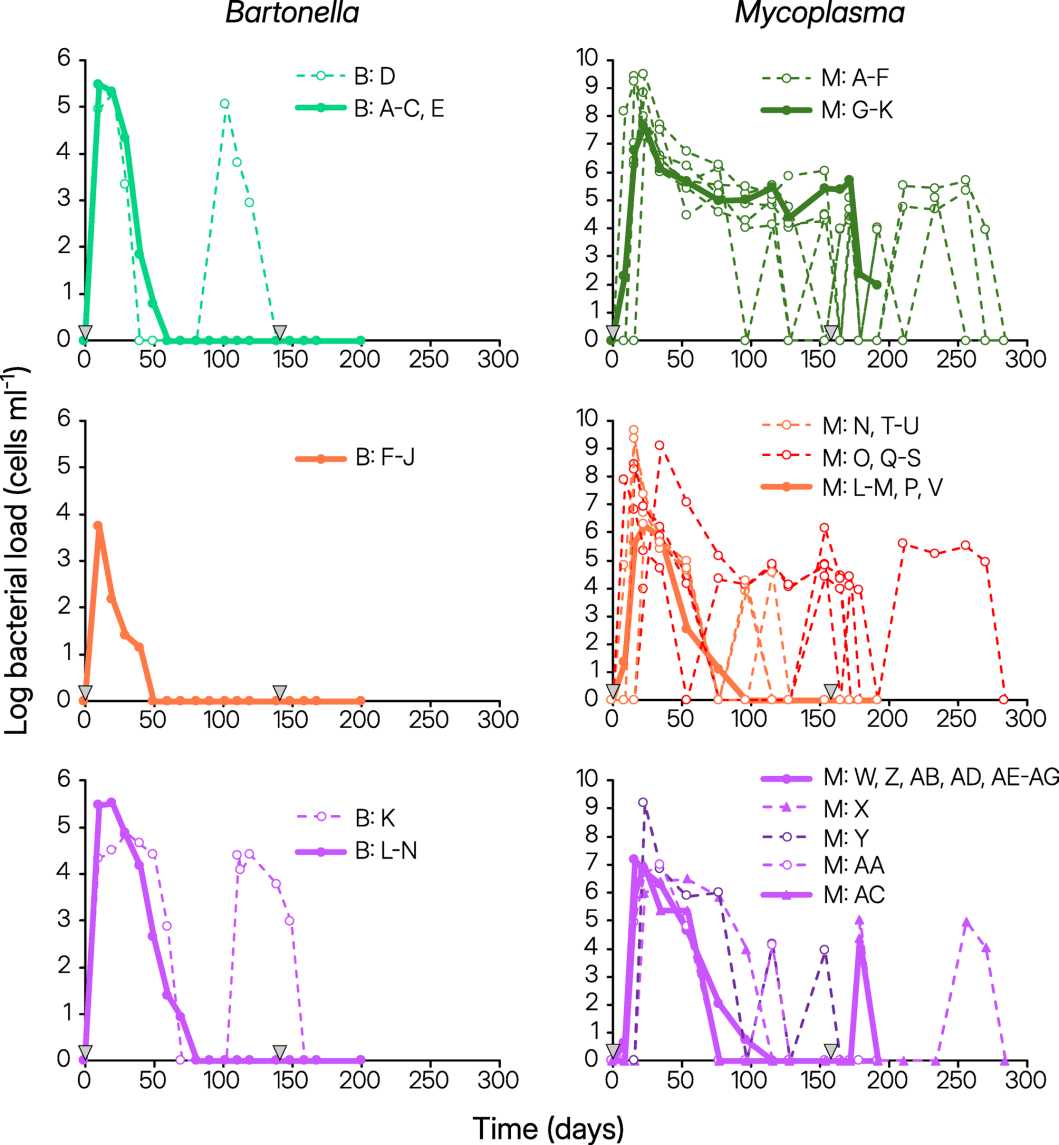
Dynamics of *Bartonella* (left panels) and *Mycoplasma* (right panels) infections in *Gerbillus andersoni* (green, upper panel), *G. gerbillus* (orange/red, middle panel), and *G. pyramidum* (purple, bottom panel) rodents. A total of five males of each rodent species were inoculated with *Bartonella krasnovii* A2 and eleven males of each species were inoculated with *Mycoplasma haemomuris-*like bacterium. The bacterial dynamics in their blood are depicted following primary and repeated inoculations. Inoculation days are denoted by grey arrows. The legends provide the specific host codes for animals inoculated with *Bartonella* (B) and *Mycoplasma* (M). Continuous infections are represented by solid lines, typically shown as means of several individuals. Recurrent infections are indicated by dashed lines, separately provided for each individual. Persistent infections, enduring throughout the entire experiment, are depicted with dark green, orange, and purple colors, while acute infections, which were cleared before the experiment concluded, are represented by light colors. Two rodent individuals that lost their *Mycoplasma* infection and exhibited a reinfection upon the repeated inoculation are marked with purple triangles (*G. pyramidum* numbers M: X and M: Y). The individual dynamics is illustrated in greater detail in Supplementary Figs. S2–S3.

Prior to commencement, we ensured that all individual rodents tested negative for *Bartonella* and *Mycoplasma* through molecular testing of their blood samples taken 1–2 weeks before inoculation. Then, following pathogen inoculations (inoculation day = day 0), blood samples were drawn from *Bartonella*-inoculated hosts every 9–11 days until day 139 post-inoculation. For *Mycoplasma*-inoculated hosts, samples were taken every 7–12 days until day 35 post-inoculation, and then every 12–26 days until day 154. All individuals subjected to the same treatment were bled on the same day. These samples were then used to extract DNA and perform a real-time quantitative polymerase chain reaction (qPCR) to measure pathogen loads. Once most rodents tested negative (days 140 and 157 post-primary inoculations for *Bartonella* and *Mycoplasma*, respectively), we re-inoculated all individuals with the original bacterium species and continued to sample their pathogen loads at least four more times (Fig. 1 and Supplementary Figs. S2−S3).

To confirm that our procedure is aseptic and that the molecular assays are specific to each of the bacterial pathogens, we also inoculated control animals on day 0. Eighteen control rodents received phosphate-buffered saline (PBS), and seventeen received pathogen-free blood, each at the same volumes used for the experimental animals. Subsequently, to confirm that the second inoculum is viable, in parallel to the repeated inoculation of the experimental animals, three of these control animals were inoculated with *Bartonella* and three with *Mycoplasma*. All control rodents were sampled alongside the experimental rodents during all blood sampling events.

The handling and experimental procedures were approved by the Committee for the Ethical Care and Use of Animals in Experiments of Ben-Gurion University of the Negev (permissions number IL‐59092015 and IL-76092019B), and the animals were housed in the Hawlena laboratory under permission from the Israel Nature and Parks Authority (number 201440611). All experiments were performed in accordance with relevant guidelines and regulations. The study is reported in accordance with ARRIVE guidelines (https://arriveguidelines.org).

### Pathogen inoculation

To prepare the *Bartonella* inoculum from the frozen *B. krasnovii* A2 stock in the Hawlena laboratory, we cultivated cells to achieve confluent growth on two chocolate agar plates. Subsequently, we harvested all cells from these plates and diluted the pathogens in 5 ml of PBS to attain a concentration of 3.2 × 10^8^ colony-forming units (CFU) per ml. This concentration was selected because, when using an inoculum volume of 0.1 ml, it ensures the minimum pathogen quantity necessary for achieving a 100% success rate in *B. krasnovii* A2 inoculations, while also aligning with the typical infestation loads observed in *Gerbillus* rodents naturally^31,32^. We then administered intradermal injections of 100 µl of the inoculum to each individual rodent, employing a 30G needle. The effectiveness of the inoculation was determined by the formation of a bleb at the injection site. We opted for intradermal injections as they closely mimic the flea-borne transmission dynamics experienced by *Bartonella* in its natural habitat^31^. We performed the inoculations under isoflurane anesthesia; immediately afterward, we returned the rodent to its cage and confirmed daily thereafter that there were no skin reactions.

Unlike *Bartonella*, *Mycoplasma haemomuris*-like bacteria cannot be cultured, necessitating the subcutaneous inoculation of hosts with blood obtained from *Mycoplasma*-positive rodents, preserved in 20% DMSO (Sigma-Aldrich, Buchs, Switzerland), and stored at − 80 °C. The inoculum was then diluted with PBS to achieve a final concentration of 1 × 10^5^ pathogenic cells (confirmed via qPCR) and a total volume of 200 µl. This concentration was selected because it falls within the typical range of pathogenic cell counts in infected rodents (10^2^–10^6^ cells per µl of blood^30^) and facilitates a significant rate of infection across the three host species while minimizing the necessary blood volume for inoculation (45 ± 8 µl of donor blood^2^). Preliminary hemagglutination tests conducted between and within rodent species showed no evidence of incompatibility. However, to mitigate any potential variations in blood donations, a block design was employed, wherein each donation was allocated to concurrently inoculate one or two host individuals from each species.

### Quantification of pathogen loads

We evaluated the pathogens loads of each rodent by collecting 50 µl of blood from the retro-orbital sinus under local anesthesia (Localin drops, Fischer Pharmaceutical LTD). We collected the blood using capillaries coated with 0.14% anticoagulant (Ethylenediaminetetraacetic acid; EDTA) and stored it in EDTA blood collection tubes (Microvette, 500 µl, SARSTEDT Group) at − 80 °C for subsequent molecular analyses. DNA extraction from the blood samples was carried out using a QIAamp BiOstic Bacteremia DNA Kit (QIAGEN, Hilden, Germany), following the manufacturer’s protocol. In each extraction session, a negative control was included, where all of the reagents were added to PBS instead of extracted blood.

Quantification of bacterial pathogen concentrations was conducted by qPCR (CFX Connect System, Bio-Rad Laboratories Inc., CA, USA), following the methodology described by Eidelman et al.^33^. For *Bartonella* load quantification, we targeted the citrate synthase (gltA) gene using 2 × qPCRbio Fast qPCR Probe Blue Mix, Hi-ROX (PCR Biosystems), 400 nmol l^− 1^ of the gltA forward primer 5ʹ GGATTTGGTCACCGAGTCTATAAA-3ʹ, 400 nmol l^− 1^ of the gltA reverse primer 5ʹ AAGAAGCGGATCGTCTTGAATAT-3ʹ, 200 nmol l^− 1^ of probe 5ʹ CCACGTGCAAAAATCATGCAAAAAACCTGTCA-3ʹ (PrimerDesign Ltd, Chandlers Ford, UK), and 2 µl of DNA in a total volume of 20 µl. The qPCR conditions comprised 3 min at 95 °C followed by 41 cycles of 10 s at 95 °C and 30 s at 60 °C. For *Mycoplasma* load quantification, the master mix was supplemented with 200 nmol l^− 1^ of the 16 S rRNA gene forward primer 5ʹ GGAGCGGTGGAATGTGTAG-3ʹ and 200 nmol l^− 1^ of the 16 S rRNA gene reverse primer 5ʹ GGGGTATCTAATCCCATTTGC-3ʹ, 100 nmol l^− 1^ of probe 5ʹ TYAAGAACACCAGAGGCGAAGGCG-3ʹ^34^, 1.5 mmol l^− 1^ of MgCl_2_, and 5 µl of DNA in a total volume of 20 µl. PCR conditions included 3 min at 95 °C followed by 35 cycles of 10 s at 95 °C and 30 s at 60 °C.

To estimate absolute copy numbers, we included, in each run, a ten-fold serial dilution standard curve of *B. krasnovii* A2 stock or previously sequenced plasmid containing the 16 S rRNA gene of *M. haemomuris*-like bacteria. The *Bartonella* standard curve was calibrated using CFU counts. To mitigate overestimation of absolute numbers by plasmid standards, the *Mycoplasma* standard curve was calibrated using Droplet Digital PCR (Bio-Rad Laboratories Inc., CA, USA), which enables direct counting of nucleic acid molecules.

### Statistical analyses

To quantify the species differences in the pathogens’ dynamics, we performed generalized linear models in which the dependent variables were the *Bartonella* or *Mycoplasma*’s (i) first day of infection detection, defined as the first day after inoculation on which the presence of each pathogen was confirmed through molecular analyses; (ii) day of peak infection; (iii) infection length (days); (iv) rate of infection increase, defined as the maximum rate of pathogenic cells’ fold change between two consecutive sampling points (cells ml^− 1^ day^− 1^); (v) recurrence probability, defined as the likelihood of the pathogen re-emerging after reaching a temporary undetectable levels during the primary infection; (vi) clearance probability of primary infection; (vii) reinfection probability, defined as the likelihood of the pathogen being reintroduced following a secondary inoculation, after the primary infection has been cleared; (viii) peak infection load resulting from the primary inoculation (cells ml^− 1^); and the (ix) overall infection load throughout the entire dynamic phase following the primary inoculation, defined as the area under the pathogen dynamics curve (AUC), normalized by the total number of days monitored (cells ml^− 1^). The host species served as the independent variable. For the probability analyses (dependent variables v–vii), we utilized binomial distributions with post-hoc comparisons between species conducted via chi-square tests. For all other analyses, we utilized Gaussian distributions, with post-hoc tests performed using Fisher’s Least Significant Difference (LSD).

All analyses were performed using R v4.1.3^35^. We computed the area under the dynamics curves using the *auc()* function within the *flux* package^36^. Additionally, we determined the maximum fold-change rate of infection increase using the *easylinear()* function in the *growthrates* package^37^, with maximum intervals set to two time points.

## Results

### General results

**Fig. 2:**
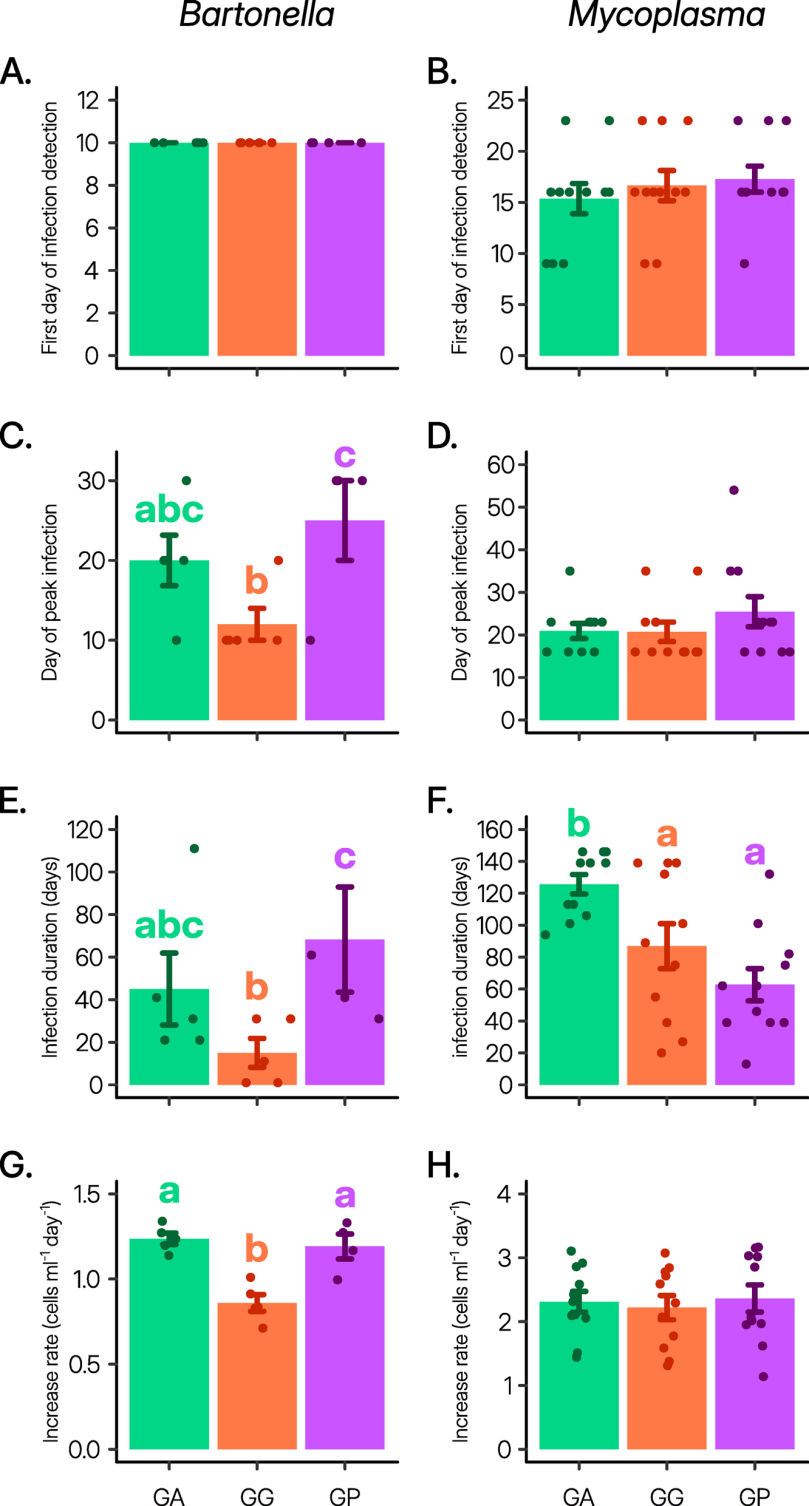
Timing and rate of infection dynamics. Means ± SE of the various parameters describing the timing and rate of *Bartonella krasnovii* A2 (left) and *Mycoplasma haemomuris*-like bacterium (right) infection dynamics in *Gerbillus andersoni* (GA, green), *G. gerbillus* (GG, orange), and *G. pyramidum* (GP, purple). Points indicate the host individual’s data, and colored letters above the bars denote significant post-hoc differences.

Overall, five males from each rodent species were inoculated with *Bartonella*, while eleven males from each species were inoculated with *Mycoplasma*. However, one *G. pyramidum* was euthanized on day 40 due to poor physical condition (individual “O” in Supplementary Fig. S3). The pathogen loads assessed were specific to the two bacterial pathogens, as evidenced by the fact that all control rodents remained pathogen-free throughout the entire experiment, except for the six control rodents inoculated during the second experimental stage. Conversely, rodents inoculated with *Bartonella* or blood containing *Mycoplasma* on day 0, became infected within 9–23 days following the primary inoculation (Figs. 1 and 2).

### Species-specific differences in pathogens dynamics

**Fig. 3:**
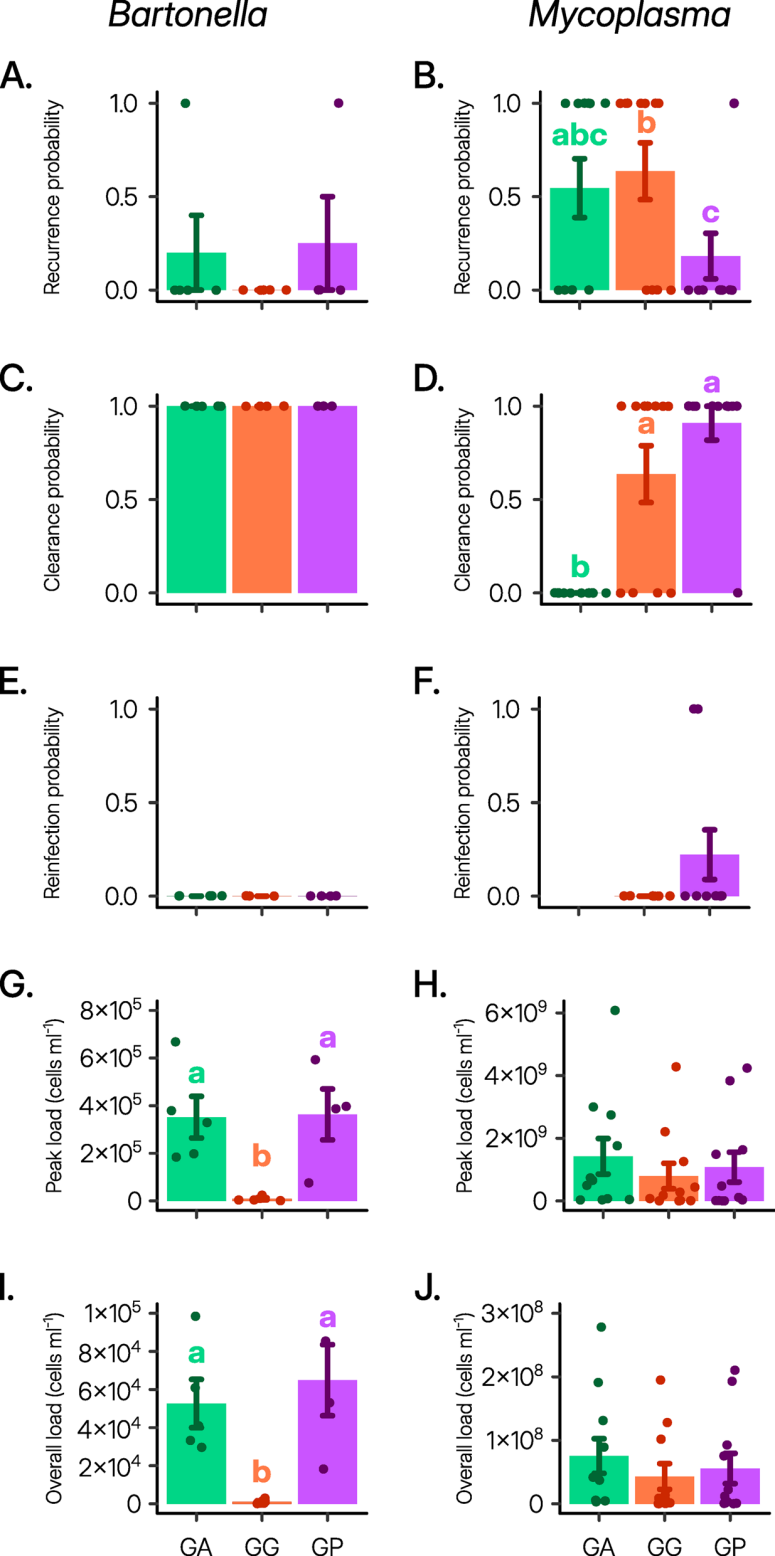
Characterization and strength of infection dynamics. Means ± SE of the various parameters characterizing the *Bartonella krasnovii* A2 (left) and *Mycoplasma haemomuris*-like bacterium (right) infections in *Gerbillus andersoni* (GA, green), *G. gerbillus* (GG, orange), and *G. pyramidum* (GP, purple) and their strength. Points indicate the host individual’s data, and colored letters above the bars denote significant post-hoc differences.

The dynamics of *Bartonella* and *Mycoplasma* within the rodent blood exhibited certain similarities (Figs. 1 and 2). First, both blood-related pathogens showed a tendency for recurrent infections (dashed lines in Fig. 1 depicting dynamics in hosts showing recurrent infections, and Fig. 3A,B). Second, the onset of infection was consistent across all three rodent species for both pathogens (Fig. 2A,B), but the duration of infection varied depending on the rodent species (Fig. 2E,F). Third, in line with the “host trait variation” hypothesis, both *Bartonella* and *Mycoplasma* pathogens demonstrated diminished performance in *G. gerbillus* compared to the other rodent species (Figs. 2E−G and 3D,F,G,I).

However, consistent with the “specific host-parasite interaction” hypothesis, all other aspects of the infection dynamics of these two blood-related pathogens exhibited varying trends across the three hosts (Table 1). First, while *Bartonella* induced acute infections across all rodent species (Figs. 1 left and 3C), *Mycoplasma*’s infection dynamics varied, ranging from acute infections in *G. pyramidum*, individual-specific dynamics in *G. gerbillus*, to chronic infections in all *G. andersoni* individuals (Figs. 1 right and 3D). Second, while the likelihood of recurrent infection by *Bartonella* did not significantly differ between the three rodent species (Fig. 3A), recurrent *Mycoplasma* infections were less frequent in *G. pyramidum* compared to the other rodent species (Fig. 3B). Third, although none of the host species became reinfected with *Bartonella* after the second inoculation, some *G. pyramidum* were reinfected by *Mycoplasma* following the second inoculation (Fig. 3E,F).

**Table 1:** Statistical results of the comparisons between the infection dynamics parameters of the three rodent species, *Gerbillus andersoni* (GA), *G. pyramidum* (GP), and *G. gerbillus* (GG). F statistics, sample size (*n*), and *p* values are provided for generalized linear models. For the probability analyses (dependent variables denoted with an asterisk), binomial distributions were employed, with subsequent post-hoc comparisons between species carried out via chi-square tests. Gaussian distributions were utilized for all other analyses, with Fisher’s least significant difference (LSD) employed for post-hoc tests. Significant differences between species are highlighted in bold. The details of the calculations for the various dependent variables are provided in the “*Statistical analyses*” section.

	*Bartonella*						*Mycoplasma*					
Dependent variable	F	n	p	Post-hoc results (*p* values)			F	n	*p*	Post-hoc results (*p* values)		
				GA vs. GG	GA vs. GP	GG vs. GP				GA vs. GG	GA vs. GP	GG vs. GP
First day of infection detection	The dependent variable has no variance						0.47	33	0.63	0.53	0.35	0.75
Day of peak infection	3.7	14	0.059	0.11	0.33	**0.022**	1.03	33	0.37	0.96	0.23	0.22
Infection duration (days)	2.5	14	0.13	0.21	0.35	**0.048**	8.93	33	**0.001**	**0.015**	**0.000**	0.119
Increase rate (cells ml^− 1^ day^1^)	17	14	**0.000**	**0.000**	0.56	**0.001**	0.15	33	0.87	0.74	0.85	0.60
*Recurrence probability	0.58	14	0.58	0.29	0.86	0.24	4.55	33	0.10	0.67	0.076	**0.030**
*Clearance probability	The dependent variable has no variance						2.04	33	0.36	**0.001**	**0.000**	0.13
*Reinfection probability	The dependent variable has no variance						0.00	16	1.0	NA	NA	0.18
Peak load (cells ml^− 1^)	7.4	14	**0.009**	**0.007**	0.917	**0.008**	0.41	33	0.66	0.37	0.62	0.69
Overall load (cells ml^− 1^)	8.0	14	**0.007**	**0.009**	0.496	**0.004**	0.46	33	0.64	0.35	0.57	0.71

Fourth, species-specific differences were absent in *Mycoplasma*’s (i) day of peak infection (Fig. 2D), (ii) maximum rate of increase (Fig. 2H), (iii) peak infection load (Fig. 3H), and (iv) overall primary infection load (Fig. 3J), whereas these variables were dependent on the rodent species for *Bartonella* (Table 1). Specifically, the day of *Bartonella*’s peak infection occurred earlier in *G. gerbillus* (Fig. 2C), and the maximum rate of increase, peak infection load, and overall primary infection load were all lowest in *G. gerbillus* compared to the other two rodent species (Figs. 2G and 3G,I). Fifth, while the *Bartonella* infection duration was shorter in *G. gerbillus* than in *G. pyramidum* rodents, the *Mycoplasma* infection duration was similarly shorter in *G. gerbillus* and *G. pyramidum* than in *G. andersoni* (Fig. 2E,F). Finally, unlike *Mycoplasma*, *Bartonella* performed similarly well in both *G. andersoni* and *G. pyramidum* rodents (Figs. 2E,F and 3C,D).

## Discussion

Parasites’ infection dynamics within hosts plays a crucial role in determining their potential transmission to other hosts, their persistence and interactions within communities, and their evolutionary trajectories^38–40^. Hence, unraveling the factors contributing to the variability in within-host parasite dynamics is a significant objective in the contexts of wildlife, domestic animals, and human health. Through inoculation experiments, we explored the infection dynamics of two bacterial pathogens cooccurring within three coexisting desert rodents. Our findings suggest that the variability in infection dynamics is not solely attributable to host heterogeneity, but rather emerges from the interplay between specific host characteristics and pathogen traits. In the following sections, we discuss the results considering the hypotheses of “host trait variation” and “host-parasite interaction,” along with their broader implications.

### Both pathogens exhibited reduced performance when infecting *G. gerbillus*

Consistent with the “host trait variation” hypothesis, both *Bartonella* and *Mycoplasma* pathogens exhibited reduced performance in *G. gerbillus* in comparison to the other rodent species. This decreased performance was evident in several aspects: a shorter infection duration, a slower rate of infection increase, and lower peak infection levels of *Bartonella* in *G. gerbillus* compared to the other rodent species; a shorter infection duration and a higher likelihood of clearance of *Mycoplasma* in *G. gerbillus* compared to *G. andersoni*; and a reduced probability of reinfection by *Mycoplasma* in *G. gerbillus* compared to *G. pyramidum*.

A key strength of this study is the longitudinal tracking of the same hosts, which revealed consistent patterns within pathogen-host combinations, despite the limited number of hosts. The consistent patterns observed across both pathogen species suggest that the variability in the aforementioned infection parameters largely stems from trait distinctions between *G. gerbillus* and the other two rodent species. All three rodent species are nocturnal, inhabit burrows, and share similar diets and predators. Moreover, *G. gerbillus* exhibits similarities in size, mean longevity, and dispersal abilities with *G. andersoni*, and microhabitat preference with *G. pyramidum* (Table S1 in ^41^). However, in the dunes of Israel’s northwestern Negev Desert, while *G. andersoni* and *G. pyramidum* persist consistently over time, *G. gerbillus* is characterized as a transient species, disappearing during years of stabilized sand and reappearing during years of shifting sand^41^. Such fluctuations in distribution may reduce the local pathogens’ adaptation to *G. gerbillus* rodents, rendering them less adept at evading or manipulating this host’s immune response. For instance, in *Schistosoma mansoni*, which have developed specific traits to survive in their snail hosts, an increase in miRNA expression was observed during interactions between the parasite and snails from the same area, compared to interactions between parasites and snails from different areas. These miRNAs target host immune genes and may be responsible for manipulating the host’s immune response^42^. The natural heterogeneity within the sand dunes of the northwestern Negev Desert, which includes distinct areas in close proximity that either host *G. andersoni* exclusively, *G. andersoni* and *G. pyramidum* together, or all three rodent species^19,41^, presents an opportunity to test this hypothesis through classical local adaptation experiments^43^. Hosts infected by pathogens from different areas, which show a shorter infection duration, a slower rate of infection increase, lower peak infection levels, greater clearance probability, and a reduced reinfection probability, compared to hosts infected by pathogens from the same area that are adapted to them, would support this hypothesis.

Alternatively, there could be physiological distinctions between *G. gerbillus* and the other two rodent hosts that render their host environment less conducive for pathogens proliferation. For instance, due to their adaptation to harsh desert conditions, *G. gerbillus* may possess higher blood osmolarity than the other two species^44^, which could potentially decelerate the growth rate of pathogens that rely on the nutrients within^45^.

### Variations in the two pathogens’ infection dynamics across the three hosts differed

Despite the reduced performance of the two bacterial pathogens in *G. gerbillus* hosts, aligning with the “specific host-parasite interaction” hypothesis, *Bartonella*, unlike *Mycoplasma*, exhibited similar infection dynamics in both *G. andersoni* and *G. pyramidum* rodents. Furthermore, unlike *Mycoplasma*, *Bartonella* demonstrated consistent acute infection, full immune protection (= no reinfection), and comparable recurrence likelihood across all three host species. These variations in host-specific effects might reflect the distinct life history strategies of the two pathogens. *Bartonella* is considered as a host-opportunistic bacterium^19^, potentially perceiving the three hosts similarly. Conversely, *Mycoplasma* exhibits a preference for infecting *G. andersoni* hosts^2,19^, potentially indicating better adaptation to consistently exploit this species. This explanation is consistent with a longstanding theory suggesting that adaptation to one environment may lead to slower adaptation and even maladaptation in foreign environments, resulting in performance trade-offs across different environments^46,47^.

The inconsistencies observed in *Mycoplasma* across hosts, compared to the persistence trends of *Bartonella*, might also stem from distinct selection pressures associated with their transmission routes between hosts. Hemoplasmas strongly rely on host-to-host contact^30^, potentially facing stronger selection pressures from the host environment than *Bartonella* ones, primarily transmitted by fleas^48^.

Conversely, several infection parameters were species-specific for *Bartonella* but not for *Mycoplasma*. These included the day of *Bartonella*’s peak infection, which occurred earlier in *G. gerbillus*, and the maximum rate of increase, peak infection load, and overall primary infection load, all of which were lower in *G. gerbillus* than in the other two rodent species. These disparities between *Bartonella* and *Mycoplasma* may indicate variations in how their adaptive immune responses are tailored to specific hosts. Rodents’ humoral immune response plays a significant role in shaping interactions with both *Bartonella*^49–51^ and hemoplasmas species^52^. However, given the suggestion that the antibody response may not play a significant role in protective immunity against hemoplasmas^53^, whereas the *Bartonella*-specific antibody response is robust, long-lasting, and associated with protective immunological memory across all three rodent species^26^, it is plausible that the impact of the humoral immune response on the timing and intensity of *Bartonella* infection is more pronounced than its effect on *Mycoplasma* infections, especially when compared to the influence of host resource quality.

### The significance of concurrently considering both pathogen and host heterogeneity

The inoculation experiments involving multiple host species and pathogens have yielded invaluable insights into pathogen dynamics within hosts, including the potential role of coadaptation in mediating infection dynamics—insights that complement those gained from single host-multiple parasites and multiple parasites-single host assays. Although the specific results may differ across parasite and host species and intraspecific groups, our findings indicate that the variation in infection dynamics across hosts may be influenced by the specific interactions between host and parasite types. This is because even parasites belonging to the same functional group may exhibit differences in life history traits; such as resource utilization efficiency, as seen in variations in clutch size and egg size among copepods infecting fish; immune evasion, with different species of parasitic fungi or nematodes using various mechanisms to survive within their hosts; and host specificity, as shown by differences in host range among parasitic copepods infecting fish^54,55^. These life history traits may, in turn, magnify or diminish the discrepancies in within-host infection dynamics across different host species.

To obtain a deeper understanding of within-host dynamics in multispecies communities, similar inoculation experiments should be further pursued in other model systems, along with experiments involving coinfection scenarios and greater intraspecific variability^11,33^. Expanding the scope to include other research systems involving closely related hosts, particularly vertebrates infected by multiple parasites from the same functional group will enable us to determine whether certain infection parameters are more host-trait specific than others. Until that point, given that the effects of host heterogeneity on parasite dynamics seem to depend on the particular interactions between hosts and parasites, it is crucial to examine each unique host-parasite interaction separately when drawing conclusions about community patterns based on infection dynamics within hosts.

## Conclusions

The objective of this study was to ascertain whether the observed variation in host responses to infections by different parasites is attributable to host-specific differences or to the manner in which parasites interact with their hosts. Our findings indicate that while both bacterial pathogens encountered difficulties in infecting a specific rodent species, the manner in which the infections manifested differed considerably between the rodents and the pathogens. This observation supports the notion that heterogeneity in infection dynamics may emerge from the interaction between host and pathogen traits. Consequently, to enhance disease control, it is imperative to examine how each pathogen interacts with each host individually.

## Electronic supplementary material

Below is the link to the electronic supplementary material.

Supplementary Material 1

## Data Availability

The datasets supporting the conclusions of this article are available in the Figshare repository and provided via 10.6084/m9.figshare.27093955.v1.
