# Supplementary material for: Variability in infection dynamics emerges from the interplay between unique host and pathogen characteristics
Source: Sci Rep. 2025 Jun 2;15:19256. doi: 10.1038/s41598-025-01351-1 (PMC12130457; doi:10.1038/s41598-025-01351-1)
Supplement: Supplementary file 1 — Supplementary Material 1 [file 41598_2025_1351_MOESM1_ESM.pdf]

Electronic Supplementary Material

Variability in infection dynamics emerges from the interplay between unique host and pathogen characteristics

Ruth Rodríguez-Pastor[1,2¶], Mario Garrido[1,3¶], Nadav Knossow[1], Naama Shahar[1], Ron Flatau[1,4], and Hadas Hawlena[1]*

[1]Mitrani Department of Desert Ecology, Swiss Institute for Dryland Environmental and Energy Research, The Jacob Blaustein Institutes for Desert Research, Ben-Gurion University of the Negev, Midreshet Ben-Gurion, Israel
[2]Present address: Agri-Food Institute of Aragon (IA2) - University of Zaragoza-CITA, Zaragoza, Spain
[3]Present address: Department of Biology and Geology, Physics and Inorganic Chemistry, Biodiversity and Conservation Area, Rey Juan Carlos University, Móstoles, Madrid, Spain
[4]Present address: Ocean Genome Legacy Center, Northeastern University, Massachusetts, USA

***Corresponding author; e-mail: hadashaw@bgu.ac.il (HH)

[¶]These authors contributed equally to this work.

Short title: Variability in infection dynamics emerges from host-parasite interplay

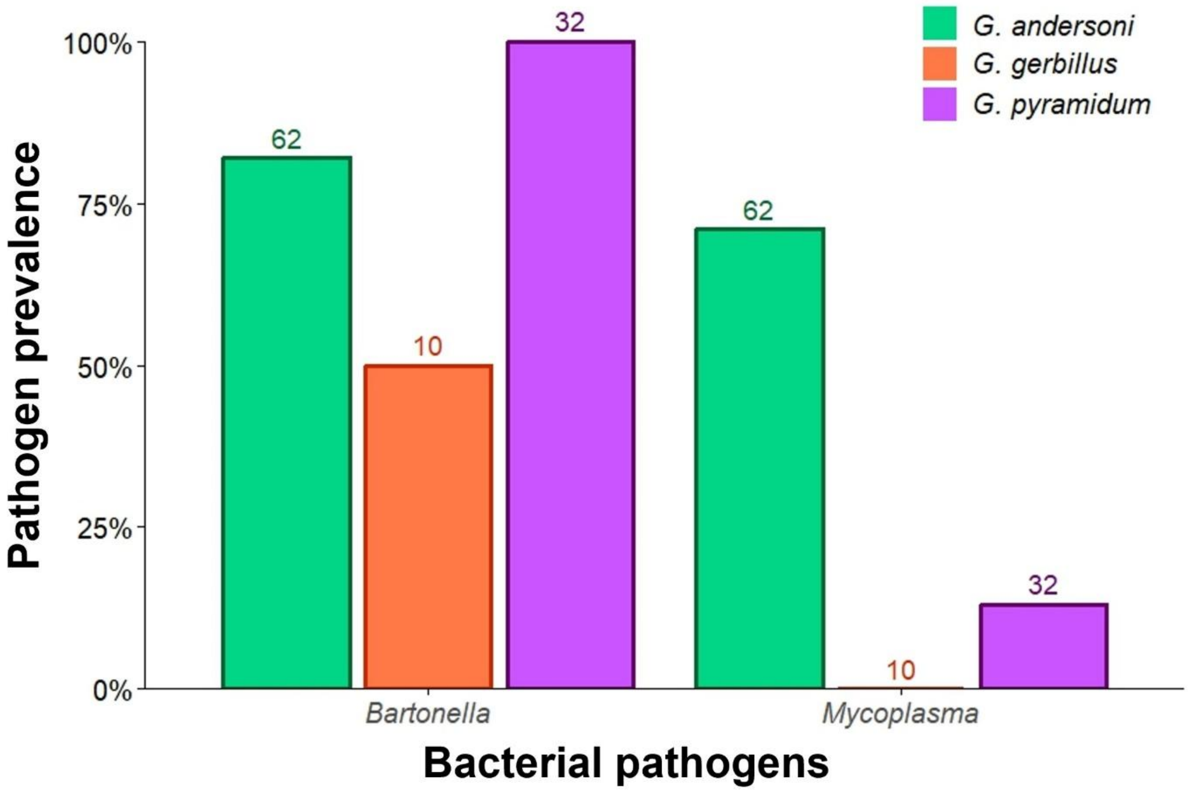

Figure S1. Prevalence of *Bartonella* and *Mycoplasma* in the dunes of Israel's northwestern Negev Desert. The bacterial prevalence values (the proportion of infected hosts) were assessed in the three coexisting rodent species: *Gerbillus andersoni* (green), *G. gerbillus* (orange), and *G. pyramidum* (purple) based on a large-scale field survey conducted in 2011. For more details, refer to Kedem et al., 2014. The numbers represent the corresponding sample sizes.

Kedem, H., C. Cohen, I. Messika, M. Einav, S. Pilosof, and H. Hawlena, *Multiple effects of host species diversity on co-existing host-specific and host-opportunistic microbes.* Ecology 2014. **95**: p. 1173-1183.

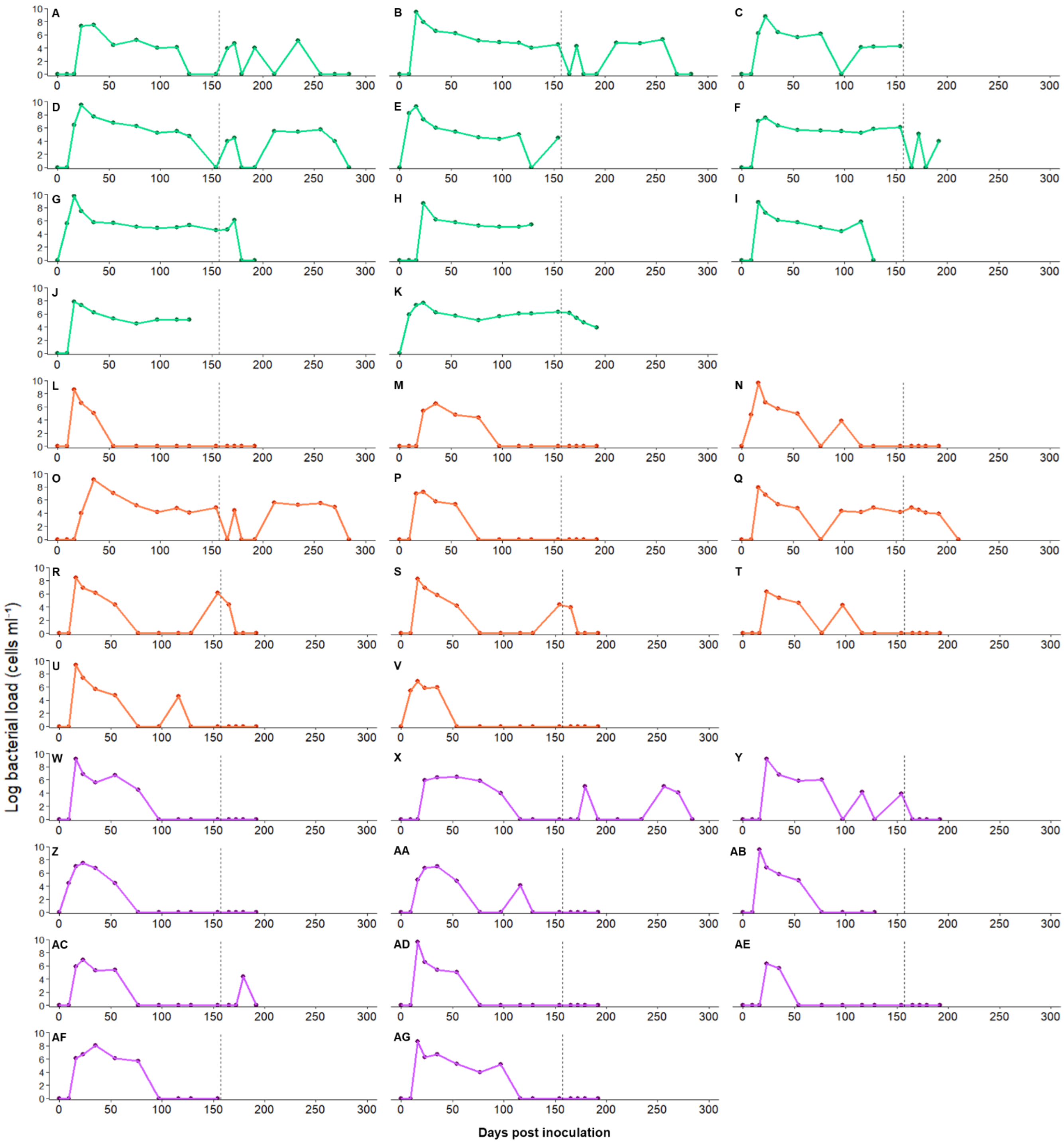

Figure S2. ***Mycoplasma*** **dynamics of individual rodents (A–AG).** Bacterial loads of 11 *Gerbillus andersoni* (green; A–K), 11 *Gerbillus gerbillus* (orange; L–V), and 11 *Gerbillus pyramidum* (purple; W–AG) were tracked in a laboratory experiment. All individuals were inoculated with *Mycoplasma*-positive blood at day 0 and then reinoculated at day 157 post inoculation (vertical dashed line). All inocula contained a final concentration of 10^5 pathogenic cells. Data are log (y + 1) transformed to facilitate comparison between species.

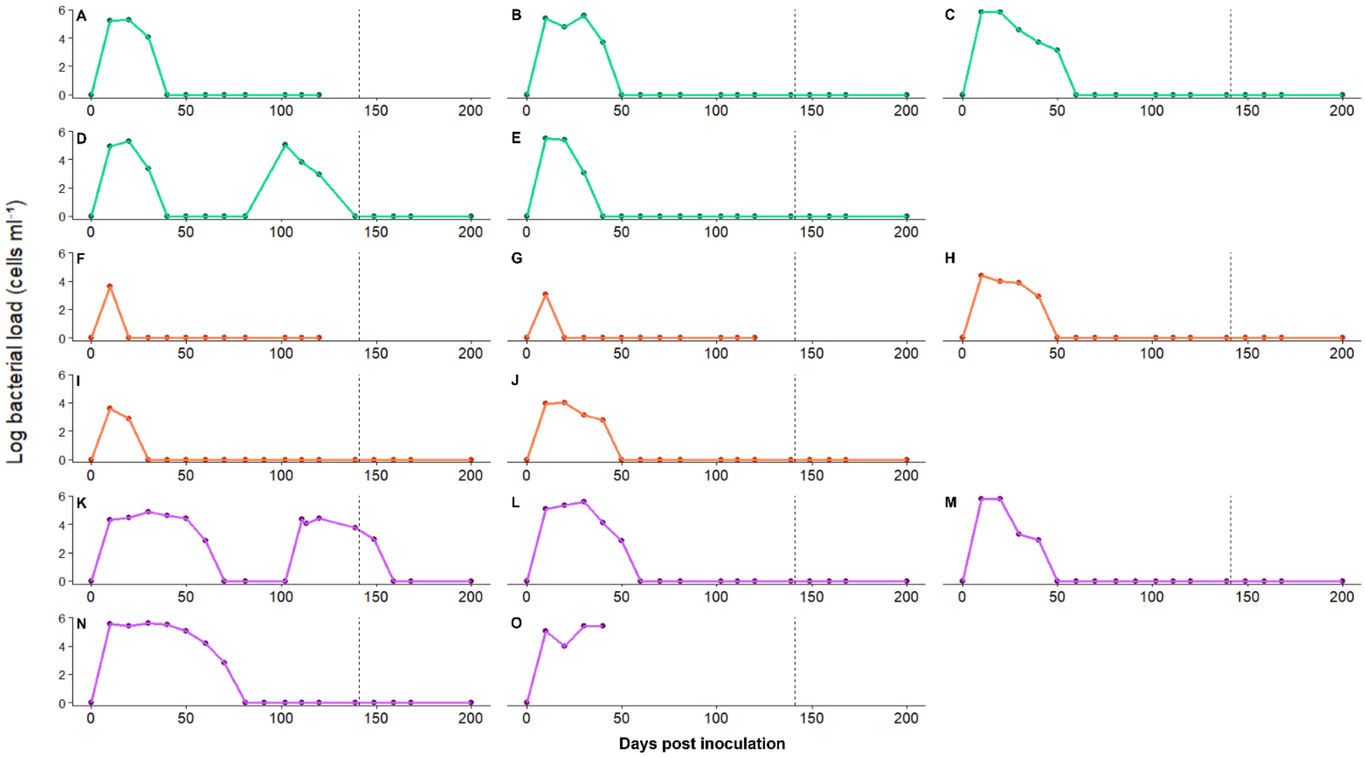

Figure S3. *Bartonella* dynamics of individual rodents (A–O). Bacterial loads of five *Gerbillus andersoni* (green; A–E), five *Gerbillus gerbillus* (orange; F–J), and five *Gerbillus pyramidum* (purple; K–O) were tracked in a laboratory experiment. All individuals were inoculated with wild-type *Bartonella krasnovii* A2 strain at day 0 and then reinoculated at day 140 (vertical dashed line). All inocula contained 10^7 colony-forming units. Data are log (y + 1) transformed to facilitate comparison between species. The bacterial dynamics in "O" is truncated because the individual was euthanized on day 40 due to its poor physical condition.
